# Chloroplast-containing coacervate micro-droplets as a step towards photosynthetically active membrane-free protocells†
†Electronic supplementary information (ESI) available: Details of experiments, microscopy data, supplementary videos, photosynthetic assay data, zeta measurements and schematic of sample holder for microscopy. See DOI: 10.1039/c8cc01129j


**DOI:** 10.1039/c8cc01129j

**Published:** 2018-03-26

**Authors:** B. V. V. S. Pavan Kumar, James Fothergill, Joshua Bretherton, Liangfei Tian, Avinash J. Patil, Sean A. Davis, Stephen Mann

**Affiliations:** a Centre for Protolife Research , School of Chemistry , University of Bristol , Bristol , BS8 1TS , UK . Email: s.mann@bristol.ac.uk

## Abstract

Encapsulation of structurally and functionally intact chloroplasts within coacervate micro-droplets is used to prepare membrane-free protocells capable of light-induced electron transport.

## Abstract 2

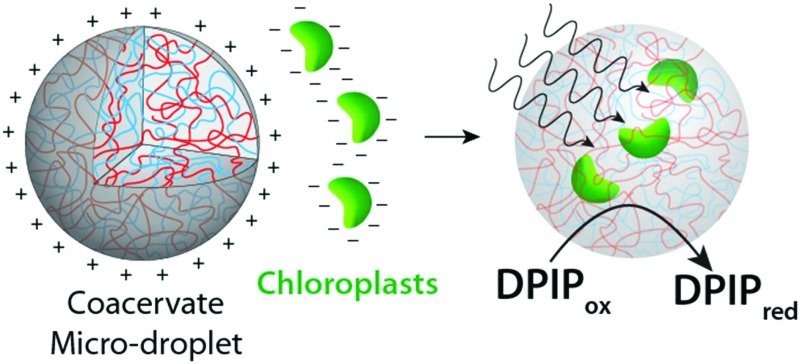
Encapsulation of structurally and functionally intact chloroplasts within coacervate micro-droplets is used to prepare photosynthetically active membrane-free protocells.

## 

*Ex situ* stabilization and integration of biological components and systems such as biomolecules, organelles and cells are important challenges for the development of new living technologies based on biocatalysis, biosensing, artificial organs, therapeutics, environmental and industrial process monitoring, and energy conversion.^1^–^4^ Stabilization of living cells in artificial constructs has received considerable attention with a range of strategies focused on the use of polyelectrolyte capsules, polymers, gels, silica and metal organic frameworks.^4^–^10^ Assimilation of biological components into membrane-bounded compartmentalized structures such as lipid vesicles,^11^,^12^ polyelectrolyte microcapsules,^13^,^14^ water-in-oil emulsions,^15^,^16^ inorganic colloidosomes,^17^ or proteinosomes^18^–^20^ has been recently explored as a step towards the construction of synthetic protocells exhibiting diverse biomimetic functionalities. Although molecular and supramolecular components such as enzymes, genetic polymers, and ribosomes have been successfully integrated into protocell models, spontaneous assimilation of microscale biological systems in the form of intracellular organelles (mitochondria, peroxisomes, chloroplasts) remains virtually unexplored.

Herein we employ a novel approach to the assimilation of biological organelles into synthetic protocells by using polymer-rich liquid micro-droplets that are spontaneously assembled in aqueous solution *via* complex coacervation.^21^ Coacervate micro-droplets have been recently investigated as molecularly crowded, membrane-free protocell models due to their ability to preferentially sequester a wide range of biomolecules and cellular machinery,^21^–^24^ promote enzymatic activity,^21^,^25^ undergo dynamical behaviour in electric fields,^26^ and act as killer protocells.^27^ In this paper, we exploit the surface and internal properties of coacervate micro-droplets prepared from bespoke mixtures of poly(diallyldimethylammonium chloride) (PDDA) and carboxymethyl-dextran (CMDX) to facilitate the sequestration and retention of intact plant chloroplasts within the membrane-free droplets as a step towards the design and construction of photosynthetically active synthetic protocells (Fig. 1). We exploit electrostatic matching between the positively charged coacervate micro-droplets and negatively charged chloroplasts to induce the capture of the organelles by surface wetting, and then employ mechanical agitation to assist transfer of the chloroplasts into the interior of the droplets. The coacervate matrix provides a stable chemical and physical environment for the uptake and retention of numerous intact chloroplasts within each droplet. We also demonstrate that the Hill reagent, 2,6-dichlorophenolindophenol (DPIP), is preferentially partitioned into the chloroplast-containing droplets such that intermittent exposures to light results in reduction of DPIP, confirming that the electron transport chain from photosystem II to plastocyanin (photosystem I) remains operational in the sequestered chloroplasts.

**Fig. 1:**
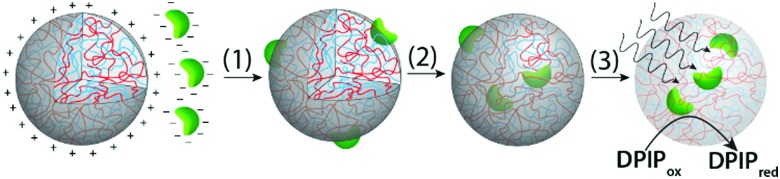
Scheme showing overall strategy for the preparation of photosynthetically active membrane-free protocells based on sequestration of negatively charged intact chloroplasts into positively charged PDDA/CMDX coacervate micro-droplets. Favorable electrostatic interactions between the micro-droplets and chloroplasts induce the facile capture of the organelles by surface wetting (1), after which the chloroplasts are gradually internalized into the coacervate interior by mechanically induced droplet coalescence and fission (2). Exposure of the chloroplast-containing coacervate droplets to light triggers the photoreduction of DPIP (3).

Chloroplasts were extracted from the leaves of *Spinacia oleracea* (spinach) and selectively isolated using discontinuous Percoll density gradients.^28^ Optical and fluorescence microscopy images indicated that the chloroplasts remained intact after extraction. The organelles exhibited a curved circular plate-like morphology with an average width and thickness of 5 and 1 μm, respectively, and displayed a characteristic green pigmentation and red fluorescence (Fig. 2a, e and Fig. S1, ESI†). The optical and fluorescence images had a distinct grainy texture, consistent with the presence of chlorophyll-containing stacked thylakoids (grana) within the intact chloroplasts. Optical absorption spectra showed peak maxima at 470 and 680 nm associated with the porphyrin metallocentre of chlorophyll *a*. As the isolated chloroplasts exhibited a net negative surface potential of *ca.* –25 mV (Fig. S2, ESI†), we used positively charged coacervate micro-droplets as host protocells for the electrostatically mediated uptake of the photosynthetic organelles. For this, coacervate dispersions were prepared from mixtures of PDDA and CMDX at an initial PDDA : CMDX monomer mole ratio of 0.3 : 1, and then centrifuged and re-dispersed in buffer to produce positively charged droplets (*ca.* +20 mV when re-dispersed in Milli Q water) (Fig. S2, ESI†). Critically, the PDDA/CMDX coacervate phase was stable across a range of buffer concentrations required to maintain the integrity of the isolated chloroplasts; in contrast, alternative formulations such as 2PDDA : 1 adenosine 5′-triphosphate (ATP) were much less stable (Fig. S3, ESI†). Optical and confocal fluorescence microscopy images of the PDDA/CMDX micro-droplets containing 5% of fluorescein-isothiocyanate (FITC) tagged CMDX showed spherical droplets with homogenous green fluorescence (Fig. 2b and f).

**Fig. 2:**
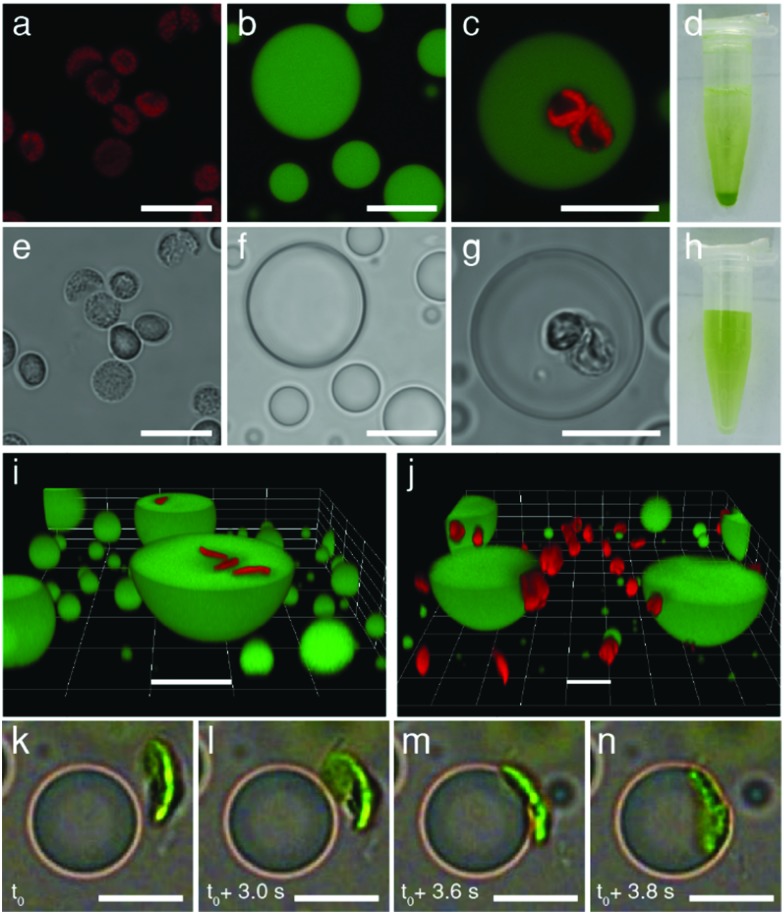
Confocal fluorescence (a–c) and bright field (e–g) microscopy images. (a and e) Isolated intact chloroplasts showing red fluorescence associated with chlorophyll-containing thylakoids (a) and grainy optical texture (e). (b and f) FITC-CMDX-doped (5%) PDDA/CMDX coacervate micro-droplets showing homogeneous green fluorescence (b) and low optical contrast (f). (c and g) As for (b and f), but showing red fluorescence and grainy optical texture within the interior of the coacervate micro-droplets due to the sequestration of two intact chloroplasts. (d and h) Photographs showing Eppendorf vials recorded after sedimentation for a few minutes of a chloroplast-containing suspension of PDDA/CMDX coacervate micro-droplets (d), or an aqueous buffered dispersion of chloroplasts (no coacervate) (h). Chloroplasts were sedimented only in the presence of the coacervate phase indicating that the organelles were strongly associated with the PDDA/CMDX micro-droplets. The green supernatant in (d) consisted of predominantly chloroplast-containing coacervate micro-droplets which were too small to sediment within a few minutes. (i and j) Confocal fluorescence microscopy images showing green fluorescent PDDA/CMDX (5% FITC-CMDX) coacervate micro-droplets after mixing with a dispersion of intact chloroplasts (red fluorescence). Mechanical agitation of the aqueous dispersions for 30 min resulted in internalization of the chloroplasts within the interior of the micro-droplets (i). In contrast, adsorbing the coacervate micro-droplets onto a glass surface prior to addition of the chloroplasts prevented droplet coalescence and fission, and gave rise to surface attachment due to interfacial wetting (j). (k–n) Time sequence of bright field microscopy images showing electrostatically meditated wetting of an approaching negatively charged chloroplast (green; viewed side-on) by a positively charged PDDA/CMDX coacervate micro-droplet. Initial attachment at one edge of the chloroplast results in complete wetting onto the droplet surface within 1 s. Samples were prepared by addition of a chloroplast suspension to a population of sedimented coacervate micro-droplets mounted onto a glass coverslip and viewed *in situ* using an optical microscope (see also, Video S2, ESI†). All scale bars, 10 μm.

Mechanically mixing aqueous buffered dispersions of the chloroplasts and a PDDA/CMDX coacervate phase resulted in sequestration of the cellular organelles into the interior of the positively charged coacervate micro-droplets (Fig. 2c and g). The corresponding microscopy images showed no changes in the optical texture or red fluorescence of the entrapped chloroplasts (Fig. 2c), which together with retention of the characteristic absorption bands (Fig. S4, ESI†) indicated that the sequestered organelles remained structurally intact within the coacervate matrix. Significantly, very few chloroplasts were observed outside the droplets and once sequestered remained irreversibly entrapped. The average number of chloroplasts captured per coacervate micro-droplet ranged from zero to several tens (Fig. S5, ESI†), depending on the volume of the droplets and the droplet/chloroplast number density ratio used to prepare the samples. The strong interaction between the intact chloroplasts (density (*ρ*) = 1.1 g mL^–1^)^28^ and positively charged PDDA/CMDX coacervate droplets (*ρ* = 1.06 g mL^–1^) was confirmed macroscopically when the mixed dispersions were vortexed and allowed to sediment for a few minutes in an Eppendorf tube to produce a green chloroplast-rich coacervate phase (Fig. 2d). In contrast, no sedimentation was observed when a dispersion of intact chloroplasts was prepared in the absence of the PDDA/CMDX coacervate under the same conditions (Fig. 2h).

3D stacked confocal fluorescence microscopy images confirmed that inclusion of the chloroplasts into the host coacervate droplets was dependent on the wetting properties of the PDDA/CMDX coacervate and mechanical agitation of the dispersions (Fig. 2i, j and Fig. S6, S7, ESI†). The former gave rise to spontaneous adsorption of the chloroplasts at the surface of the PDDA/CMDX droplets, while the latter was required to breach the viscous coacervate/water interface to produce the internalized organelles. In general, complete wetting of the chloroplasts occurred within 1–2 minutes after addition of the organelles to a dispersion of PDDA/CMDX coacervate micro-droplets and was mediated by electrostatic interactions between the colloidal objects. Optical and fluorescence microscopy studies indicated that the rate of wetting was dependent on the geometry of the interacting surfaces such that interactions involving geometrically complementary surfaces maximized the contact surface area usually within a few seconds (Fig. 2k–n and Video S1, ESI†). Alternatively, geometrical mismatches at the contact interface gave rise to slow conformal wetting that involved distortion of the coacervate droplet surface (Fig. S8, ESI†), followed by remodeling of the interface and reorientation of the attached chloroplast (Video S2, ESI†). In contrast, mixing the chloroplasts with a dispersion of negatively charged coacervates prepared at a PDDA : CMDX monomer mole ratio of 1 : 20 and surface potential of –30 mV (Fig. S9, ESI†) resulted in minimal wetting and negligible adsorption of the organelles onto the droplet surface (Fig. S10 and Video S3, ESI†).

Having established the conditions required for internalization of structurally intact chloroplasts into the membrane-free PDDA/CMDX protocells, we investigated the photosynthetic activity of the encapsulated organelles (Fig. 3a). Coupled electron transport across photosystem II and I was assayed by adding DPIP (Hill reagent) (Fig. 3b and Fig. S11, ESI†) to a buffered suspension of free chloroplasts, or to a dispersion of chloroplast-containing coacervate micro-droplets. The dispersions were subjected to a series of brief exposures (10 s) to 40 kilolux of light and the reduction of DPIP_ox_ monitored spectroscopically. Time-dependent UV-visible spectra showed that the entrapped chloroplasts reduced DPIP_ox_ at a similar rate to chloroplasts dispersed in aqueous buffer, indicating that the photosynthetic activity of the chloroplasts was retained within the polymer-rich coacervate interior (Fig. S12 and S13, ESI†). Statistically, chloroplasts encapsulated within the coacervates performed marginally better than in free buffer at low concentrations of DPIP (22.5 μM) than at higher concentrations, particularly as the number of exposures to the light was increased (Fig. 3c and Fig. S14, ESI†). We attributed this to the preferential concentration of DPIP_ox_ in the PDDA/CMDX coacervate phase as evidenced by the coloration of the centrifuged samples (Fig. 3d), and a measured partition coefficient of 3.3 ± 0.08. However, because the Hill reaction was weakly dependent on DPIP_ox_ concentration (Fig. S15, ESI†) only marginal increases in activity were observed for the chloroplast-containing coacervate micro-droplets. Degradation assays using the Hill reaction were conducted periodically every 24 h over a few days to assess the potential of the PDDA/CMDX coacervate micro-droplets as longer-term hosts for the chloroplasts. Chloroplasts internalized into the coacervates were found to exhibit a half-life of around 3 days, which was similar to the half-life determined for buffered suspensions of isolated chloroplasts (Fig S16, ESI†).

**Fig. 3:**
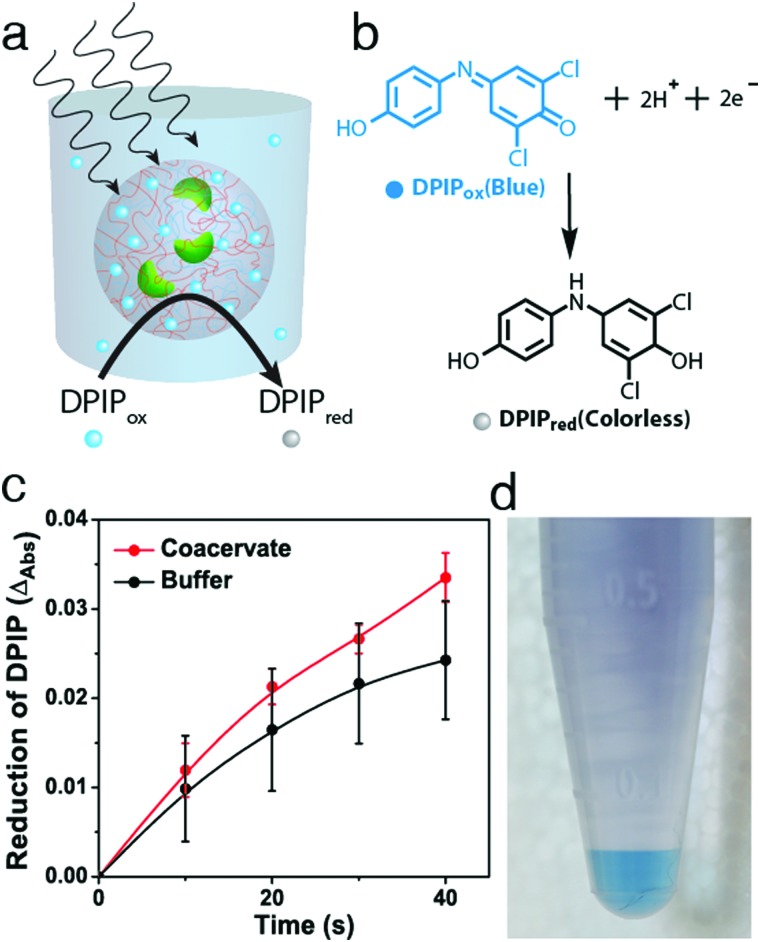
(a) Graphic showing light-induced reduction of DPIP_ox_ (blue) to DPIP_red_ (colorless) by photosynthetic activity of chloroplasts sequestered within a PDDA/CMDX coacervate micro-droplet. (b) Corresponding reaction scheme for DPIP reduction. (c) Plots showing the reduction of DPIP (22.5 μM) with time associated with the photosynthetic activity of chloroplasts internalized into PDDA/CMDX coacervate micro-droplets (red) and dispersed in buffer (0.33 M d-sorbitol, 50 mM HEPES, 2 mM Na–EDTA, 1 mM MgCl_2_, pH 7.5) in the absence of the coacervate (black). Aliquots were analysed by UV-spectroscopy after brief exposures (10 s) of the dispersions to 40 kilolux of light. Dispersions were stored in the dark on an ice bath between light exposures. (d) Photograph showing a centrifuged suspension of PDDA/CMDX coacervate micro-droplets prepared in the presence of DPIP_ox_. The presence of a blue lower DPIP_ox_-rich PDDA/CMDX coacervate layer was associated with preferential sequestration of DPIP_ox_ into the coacervate phase.

Finally, we employed an acoustic standing wave pressure field^29^ to generate 2D arrays of the chloroplast-containing coacervate micro-droplets supported on a glass substrate. This was achieved by adding a mixture of freshly prepared PDDA/CMDX coacervates and chloroplasts into the acoustic device and spontaneously trapping the organelle-containing droplets under water on a glass substrate at the nodes of the acoustic pressure field. The *in situ* patterning procedure gave rise to a regular 2D array of discrete protocells with a uniform inter-droplet centre-to-centre spacing of 110 μm, which was equal to half the wavelength of the acoustic standing wave pressure field (Fig. 4a). As the droplets were assembled and trapped in the presence of the acoustic field, the mean droplet size was highly uniform (mean size, 67 ± 8 μm) compared with analogous samples produced in free solution. As a consequence, the number distribution of chloroplasts per protocell was more uniform in the trapped droplets with a mean value of *ca.* 8 organelles per droplet (Fig. 4b). Moreover, compared with the coacervate dispersions (Fig. S5, ESI†), virtually all the chloroplasts were located within or on the surface of the trapped droplets (Fig. 4b).

**Fig. 4:**
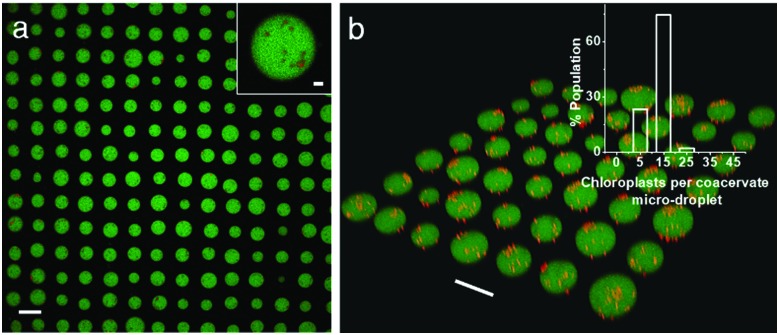
(a) Confocal fluorescence microscopy image showing acoustically trapped 2D geometric array of chloroplast-containing PDDA/CMDX (5% FITC-CMDX, green fluorescence) coacervate micro-droplets. The droplets are *ca.* 70 μm in mean size and patterned into a square grid-like lattice with uniform centre-to-centre spacing (*ca.* 110 μm). Inset; magnified image of a single coacervate micro-droplet in the array containing several chloroplasts (red). Scale bars, 100 μm and 10 μm (inset). (b) Corresponding 3D confocal fluorescence microscopy image of the spatially trapped chloroplast-containing protocells; scale bar, 100 μm. Inset showing the number distribution of chloroplasts associated with acoustically trapped arrays of coacervate micro-droplets. The majority of the micro-droplets contain 6–15 chloroplasts per micro-droplet.

In conclusion, we demonstrate that controlling the surface potential of coacervate micro-droplets can be used to capture intact plant chloroplasts to produce a membrane-free protocell model capable of light-induced electron transport. Up to several tens of chloroplasts can be encapsulated into each protocell by a combination of electrostatically mediated wetting and gentle mechanical agitation. The chloroplasts retain their structural and functional integrity after immobilization within the coacervate phase and remain photoactive with a similar half-life of 3 days compared with isolated chloroplasts in buffer. Significantly, the chloroplast-containing coacervate micro-droplets can be acoustically patterned into periodic 2D arrays with a controllable spacing, well-defined droplet size and narrow number distribution of the guest organelles.

From a wider perspective, given the current need for clean energy and carbon dioxide remediation, chloroplasts are an attractive photoactive target to encapsulate or stabilize in *ex situ* environments. Indeed, chloroplasts have been stabilized in silica-based inert matrices for use as artificial photosynthetic bioreactors,^30^–^32^ and the extension to dispersed soft matter systems such as coacervates and vesicles^33^ could provide new opportunities in flow-based systems and patterned droplet arrays. In the latter case, the ability to spontaneously produce uniform arrays of droplet-isolated chloroplasts might provide a novel route to photosynthetically active micro-chips for biomimetic water splitting.

We thank the EPSRC, ERC Advanced Grant Scheme (ERC-2016-ADG 740235), Bris-SynBio, Marie-Curie Individual Fellowship (B. V. V. S. P. K., MSCA-IF-2015-EF 705165) and University of Bristol (A. J. P. and S. A. D.) for financial support and James Ashbolt for his assistance with preliminary proof of principle experiments.

## Conflicts of interest

There are no conflicts to declare.

## Supplementary Material

Supplementary information

Supplementary movie

Supplementary movie

Supplementary movie
