# Supplementary material for: Chloroplast-containing coacervate micro-droplets as a step towards photosynthetically active membrane-free protocells
Source: Chem Commun (Camb). 2018 Mar 26;54(29):3594–7. doi: 10.1039/c8cc01129j (PMC5885784; doi:10.1039/c8cc01129j)
Supplement: Supplementary file 1 [file CC-054-C8CC01129J-s001.pdf]

Electronic Supplementary Material (ESI) for ChemComm.
This journal is © The Royal Society of Chemistry 2018

Supporting Information

Chloroplast-containing coacervate micro-droplets as a step towards photosynthetically active membrane-free protocells

B.V.V.S. Pavan Kumar, James Fothergill, Joshua Bretherton, Liangfei Tian, Avinash J. Patil, Sean A. Davis and Stephen Mann*

Centre for Protolife Research and Centre for Organized Matter Chemistry, School of Chemistry, University of Bristol, Bristol BS8 1TS, UK.

MATERIALS and METHODS

Materials: All materials were used without further purification. Poly(diallyldimethylammonium chloride) (PDDA, M_W ≈ 200 – 350 kDa), carboxymethyl-dextran sodium salt (CMDX, M_W ≈ 10-20 kDa), bovine serum albumin (BSA), fluorescein isothiocyanate-carboxymethyl-dextran (FITC-CMDX, M_W ≈ 70 kDa) D-sorbitol, 4-(2-hydroxyethyl)-1-piperazineethanesulfonic acid (HEPES), ethylenediaminetetraacetic acid disodium salt dehydrate (Na-EDTA), magnesium chloride hexahydrate, 2,6-dichloroindophenol (DPIP), and polyethylene glycol (M_w ≈ 8000) were obtained from Sigma. Percoll™ was obtained from Fischer Scientific and 2-methoxy(polyethyleneoxy)propyl trimethoxysilane (PEGsilane, 90%) was obtained from ABCR GmbH & Co. KG. Milli-Q water (18.2 MΩ·cm) was used in the preparation of all aqueous solutions.

Optical and confocal fluorescence microscopy: Optical microscopy experiments were carried out on a Leica DMI 3000B optical microscope. Fluorescence imaging was performed using a Leica DFC 310FX set up, and chloroplasts were excited by using a filter with the following excitation (λ_{ex}) and emission wavelength (λ_{em}) cut offs: λ_{ex} = 515 - 560 nm and λ_{em} = 580 nm. Confocal fluorescence microscopy measurements were performed using a Leica SP5-II AOBS confocal laser scanning microscope attached to a Leica DM I6000 inverted epifluoresence microscope, and equipped with a ×100 objective (1.4 NA). 3D reconstructions were processed with Icy software.

Extraction of chloroplasts from spinach (*Spinacia oleracea*): 15 g of leaves of spinach (Sainsbury's, UK) were stored in the dark at 4°C for 1-2 days and then cut into small pieces after removing the midribs, crushed using a mortar-pestle with 30 mL of ice cold grinding buffer (0.33 M D-sorbitol, 50 mM HEPES, 2 mM Na-EDTA, 1 mM $MgCl_2$, pH 7.5), and filtered through 4 layers of miracloth. The filtrate was centrifuged at 300 x g for 60 s to sediment any intact plant cells. The supernatant was further centrifuged at 2000 x g for 10 min to isolate the extracted chloroplasts as a pellet, which was then gently resuspended in 3-4 mL of grinding buffer using a brush. Rapid purification of the plastids was undertaken using discontinuous Percoll gradients.[1] For the gradient preparation, 3 % (w/v) polyethylene glycol 8000 and 0.1 % (w/v) bovine serum albumin (BSA) were dissolved in the Percoll™ (Fischer) solution (Percoll-PEG-BSA medium). Gradient solutions were made up by mixing different volumes of Percoll-PEG-BSA medium with a 5-fold concentrated grinding buffer. The discontinuous gradient was prepared in 50 mL centrifuge tubes, and consisted of a bottom layer (10 mL, 80% Percoll-PEG-BSA), a middle layer (20 mL, 45% Percoll-PEG-BSA)

and an upper layer (5 mL, 10% Percoll-PEG-BSA). 3-4 mL of the resuspended chloroplasts were loaded on top of the Percoll density column, and the samples centrifuged at 10000 x g for 5 min. Intact chloroplasts accumulated at the 45-80 % interface, and were isolated and mixed with an equal volume of grinding medium, and then pelleted by centrifugation (60 s at 1500 x g) to remove the Percoll. The sediment was washed three times by re-suspending the intact chloroplasts in grinding buffer followed by centrifugation, and then stored in the grinding buffer at 4°C.

Estimation of chlorophyll content was carried out using the Arnon method.[2] In brief, 0.5 mL of the chloroplast suspension was diluted with 10 mL of 80 % v/v acetone-water solution and the absorbance measured at 645 nm and 663 nm using a Perkin Elmer Lambda750 UV/Vis spectrophotometer. The chlorophyll content was calculated using the equation:

$$chlorophyll\ (mg\ mL^{-1}) = (20.2\ \times\ A_{645}\ +\ 8.02\ \times\ A_{663})\ \times\ \frac{V_{total}}{1000\ \times V_{chloroplast\ suspension}}$$

where, A_{645} and A_{663} refer to the absorbance at 645 nm and 663 nm, respectively, and V_{total} = volume of the chloroplast solution + the volume of 80 % v/v acetone-water solution.

Sequestration of chloroplasts into PDDA/CMDX coacervate micro-droplets: Positively-charged coacervate micro-droplet dispersions were prepared by mixing 0.125 mL of poly(diallyldimethylammonium chloride) (PDDA, 400 mM) and 0.45 mL of carboxymethyl-dextran (CMDX, 370 mM), and the volume was made up to 1 mL using grinding buffer. The coacervate phase was isolated by centrifugation and redispersed in the grinding buffer maintaining the volume of the dispersion. The chloroplast suspension (0.45 mg mL^{-1} chlorophyll, 37.5 µL) was added directly or after dilution to 0.5 mL with the grinding buffer to a coacervate micro-droplet dispersion in grinding buffer (0.5 mL). The dispersions were then placed on a vortexer for 20 minutes, and then observed using bright field and fluorescence microscopy. 3D stacks of fluorescence images were acquired using confocal microscopy to establish the inclusion of chloroplasts into the micro-droplets. To enable visualization of the coacervate phase using fluorescence/confocal microscopy, 5% of FITC tagged CMDX was added to the coacervate mixture.

Electrostatically mediated interactions between the coacervate micro-droplets and chloroplasts were imaged in a custom-built sample holder (Supplementary Fig. S16). 10 µL of positively charged PDDA/CMDX coacervate micro-droplets (monomer mole ratio PDDA : CMDX = 0.3 : 1) or negatively charged PDDA/CMDX coacervate micro-droplets (monomer mole ratio PDDA : CMDX = 1 : 20) were introduced into one side of the sample holder and allowed to settle onto the PEGylated glass coverslip. After *ca*. 5 minutes, 10 µL of the chloroplast dispersion were injected from the other side of the sample chamber, and the coacervate droplets then imaged during mixing with the chloroplasts.

Photosynthetic activity of chloroplasts: Assays were undertaken on dispersions of chloroplast-containing PDDA/CMDX coacervate micro-droplets (16.8 or 22.4 µg mL^{-1} chloroplasts; 1 mL, 50 mM PDDA; 165 mM CMDX) in the presence of DPIP (22.5, 30, 45 or 60 µM) by aliquot analysis of samples after a series of brief exposures (10 s) to 40 kilolux of light (Osram Ultra-Vitalux, 300 W). The reaction dispersions were stored in the dark on an ice bath between light exposures. Aliquots (100 µL) were mixed with sodium chloride

solution (200 µL, 2 M) to disassemble the coacervate phase to avoid scattering interference associated with the coacervate dispersions. Reduction of oxidized DPIP was monitored by UV-visible spectroscopy at intervals of 10 s using the time-dependent decrease in the absorption intensity measured at 620 nm. Contributions to the absorbance at 620 nm arising from the chloroplasts were corrected for by reference to the chloroplast absorption at 850 nm where DPIP showed no absorbance. All assays were carried out in glass vials treated with PEGylated silane.

Assays were also undertaken on buffered dispersions of free chloroplasts (30 µg mL^{-1}), or in the presence of individual coacervate components (50 mM PDDA or 50 mM CMDX), by adding DPIP (25 µM) and exposing the chloroplast suspensions to brief exposures (10 s) to 40 kilolux of light. Reduction of DPIP was monitored by UV-vis spectroscopy at intervals of 10 s. Different concentrations of DPIP (7.5, 10, 22.5 or 60 µM) were investigated at a constant chloroplast concentration of 5.8 µg mL^{-1}.

Degradation/stability assays were carried out by batch analysis of samples stored in the dark at 4 °C and analyzed at 48 h intervals for residual photosynthetic activity. Activity assays were performed on buffered dispersions of chloroplasts-containing PDDA/CMDX coacervate micro-droplets (11.8 µg mL^{-1} chloroplasts; 1 mL, 50 mM PDDA; 165 mM CMDX) and on free chloroplasts (11.0 µg/mL) in the presence of DPIP (22.5 µM) by aliquot analysis of samples after three 30s exposures to 40 kilolux of light (Asahi Spectra Max-303; Visible bandwith). The reaction dispersions were stored on ice between exposures to the light.

Acoustic patterning of chloroplast-containing PDDA/CMDX coacervates micro-droplets: The chloroplast-containing coacervate micro-droplet array was prepared in a custom-built acoustic trapping device with a square arrangement of four piezoelectric transducers (Noliac, NCE 51, L15 x W2 x T1 mm). The opposing transducer pairs were wired in parallel, driven by two signal generators (Agilent 33220a-001), and each connected to an oscilloscope (Agilent DSOX2014A). A glass coverslip was attached with adhesive to the bottom of the device. The two-orthogonal acoustic standing waves were generated from opposing transducer pairs operating at 6.703/6.717 MHz (10 V). The device chamber was filled with 800 µL of buffer and then 75 µL of a mixture containing freshly prepared coacervate micro-droplets and chloroplasts (50 mM PDDA; 165 mM CMDX (5% FITC-CMDX); 9.4 µg/mL chloroplasts) was added to it and mixed gently with a pipette. After *ca*. 10 min, the supernatant containing very small coacervate micro-droplets and uncaptured chloroplasts was exchanged with buffer. 3D stacks of fluorescent microscopy images of the chloroplast-containing coacervate protocell arrays were recorded to determine the number distribution of chloroplasts per coacervate micro-droplet in the patterned arrays.

Determination of DPIP partition coefficient in PDDA/CMDX coacervates: 1 mL of a PDDA/CMDX coacervate dispersion (50 mM PDDA, 165 mM CMDX) was centrifuged, and 100 µL of the supernatant replaced with DPIP solution (0.3 mM) and vortexed for 1 min to redisperse the coacervate phase in the presence of DPIP. The coacervate dispersion was then allowed to age for a few hours before analyzing the supernatant and bulk coacervate phase for the amount of DPIP partitioned between the two phases using UV-visible spectroscopy. Aliquots of the supernatant (200 µL) and coacervate phase (20 µL) were mixed with 200 µL of phosphate buffer (0.1 M, pH 7.5) and 200 µL of sodium chloride solution (1.5 M) for the UV-Vis measurements.

PEGylation of coverslips and vials. The vials and cover slips were treated with piranha solution (3 parts sulfuric acid and 1 part hydrogen peroxide) for about 1 hour, washed thoroughly with water and blow dried. A solution of PEGsilane in toluene (2% v/v) was used for surface functionalization. After about 1 hour, the vials and cover slips were rinsed with ethanol and blow dried.

Zeta potential measurements. All measurements were performed using a Malvern Zetasizer Nano-ZS instrument equipped with an internal Peltier temperature controller. The measurements were carried out in a disposable zeta cuvette at 25 °C.

Supplementary videos

Supplementary video 1. Optical microscopy video showing electrostatically-mediated capture of a single negatively charged chloroplast by a positively charged PDDA/CMDX coacervate micro-droplet. The video is shown in real time.

Supplementary video 2. Optical microscopy video showing the gradual re-structuring of the surface of a positively charged PDDA/CMDX coacervate micro-droplet on contact with a negatively charged chloroplast exhibiting conformal wetting behaviour. The video is shown in real time.

Supplementary video 3. Optical microscopy video showing the absence of interaction between a negatively charged chloroplast and a negatively charged PDDA/CMDX coacervate micro-droplet. Even after multiple collisions of the chloroplast with the coacervate micro-droplet there was no interaction or attachment observed. The video is shown in real time.

SI Figures

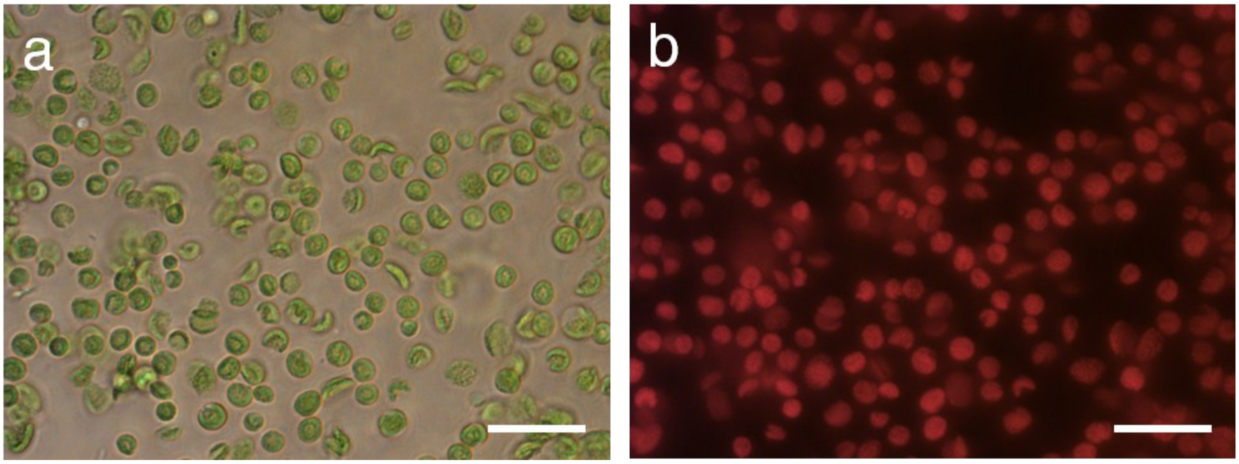

Figure S1. (a) Optical and (b) fluorescence microscopy images of isolated intact chloroplasts suspended in buffer. The grainy texture superimposed on the green pigmentation and red fluorescence confirms the presence of intact chlorophyll-containing grana (thylakoids) within the chloroplast envelope. All scale bars, 20 μm.

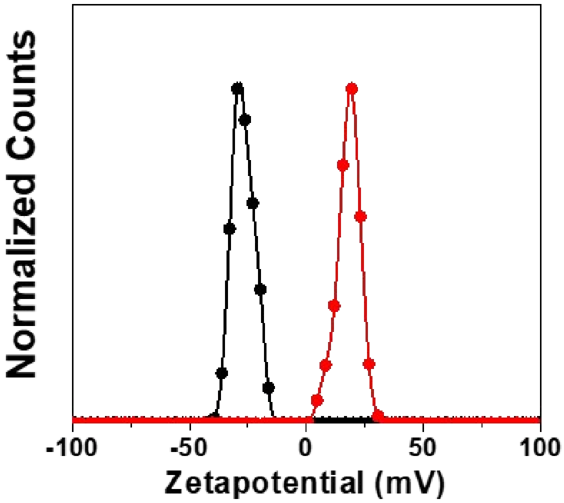

Figure S2. Zeta potential measurements for isolated intact chloroplasts (black) and PDDA/CMDX coacervate micro-droplet dispersions (red) prepared from a monomer mole ratio of 0.3 : 1; respective values of approximately -25 mV and +20 mV were determined.

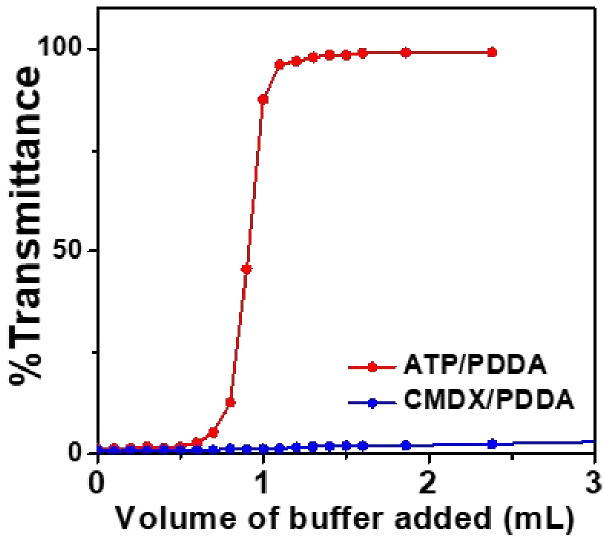

Figure S3. Plot showing change of transmittance of coacervate dispersions (1 mL) upon successive addition of the grinding buffer; the PDDA/CMDX coacervate (blue) was significantly more stable against dilution compared with a PDDA/ATP coacervate (red).

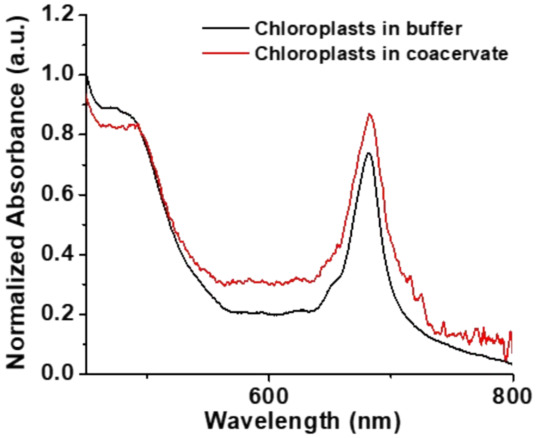

Figure S4. Absorption spectra of chloroplasts recorded in buffer or in a bulk PDDA/CMDX coacervate phase. Absorption bands in the blue (soret band, *ca.* 470 nm) and red (Q band, *ca.* 680 nm) regions were associated with π–π* transitions of the Mg porphyrin metallocentres of chlorophyll. No changes in the peak maxima were observed, indicating that the chlorophyll-containing thylakoids were not denatured upon sequestration of the chloroplasts into the coacervate phase.

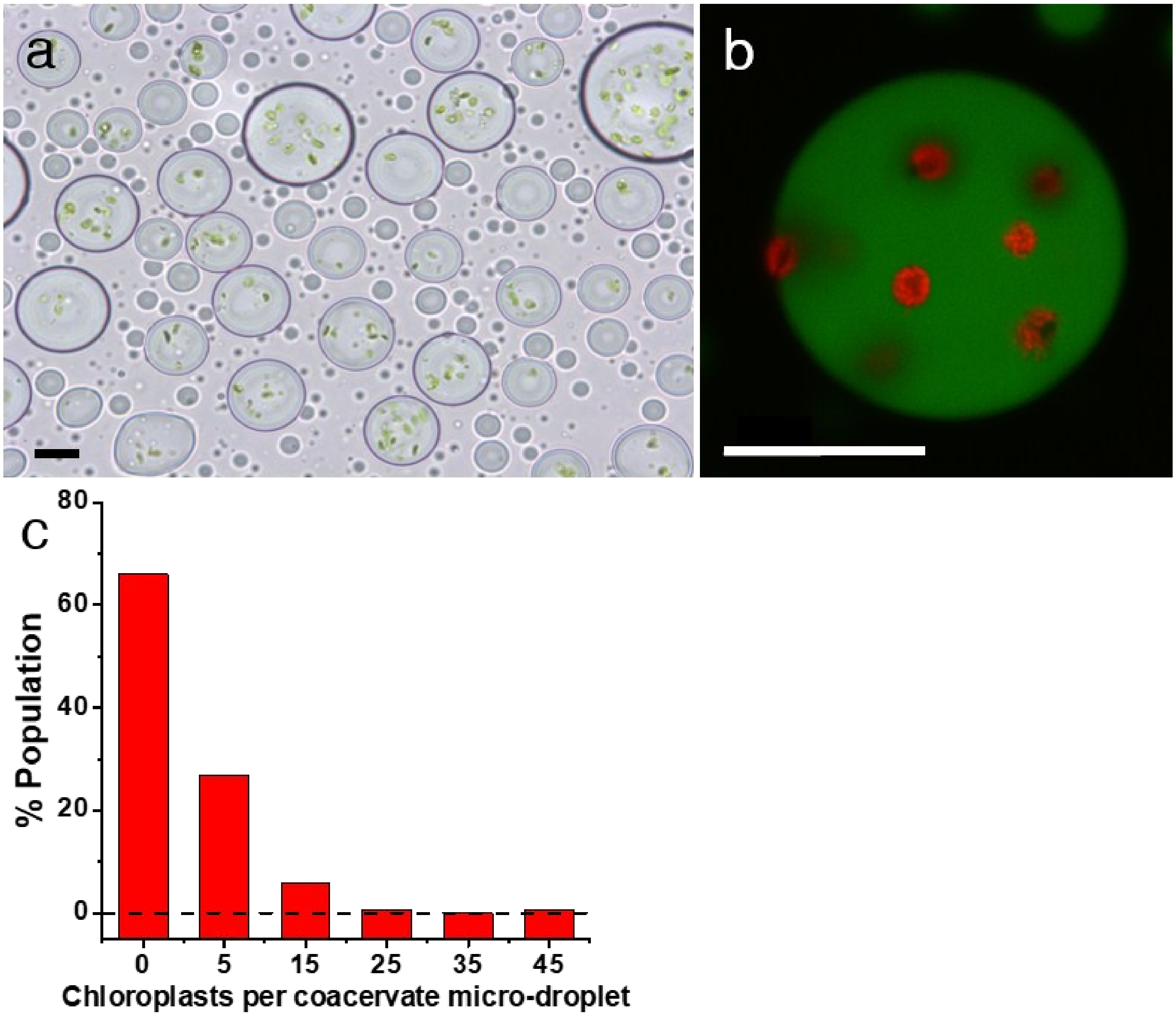

Figure S5. (a) Optical and (b) fluorescence microscopy images showing coacervate micro-droplets containing internalized chloroplasts. (c) Number distribution of chloroplasts associated with the coacervate micro-droplets. All scale bars, 20 µm.

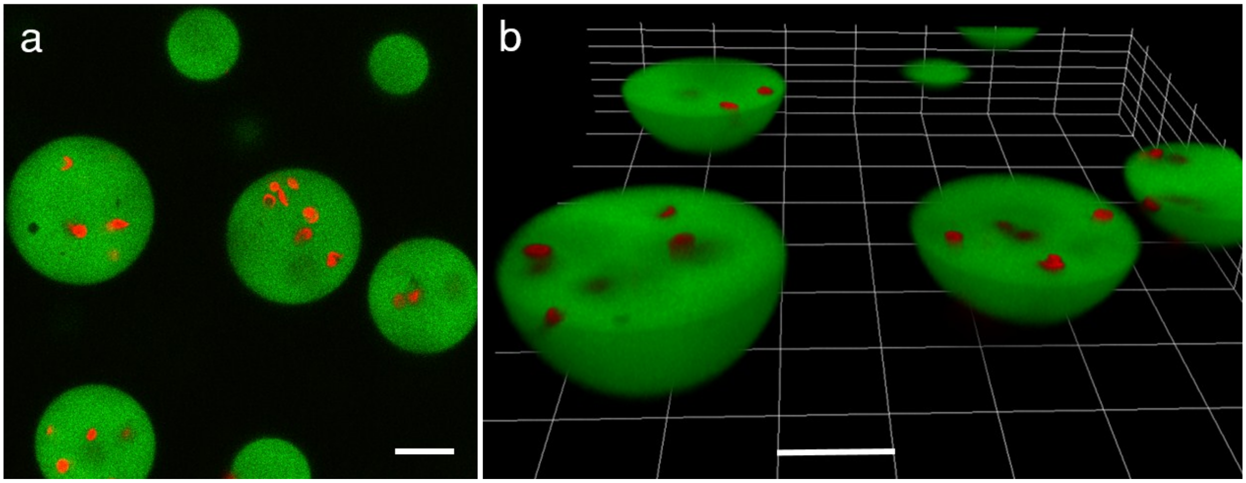

Figure S6. Confocal fluorescence microscopy images (a,b) showing sequestration of chloroplasts within the interior of PDDA/CMDX (5 % FITC-CMDX) coacervate micro-droplets. Samples were prepared by mechanically mixing the dispersions for 30 min. All scale bars, 20 µm.

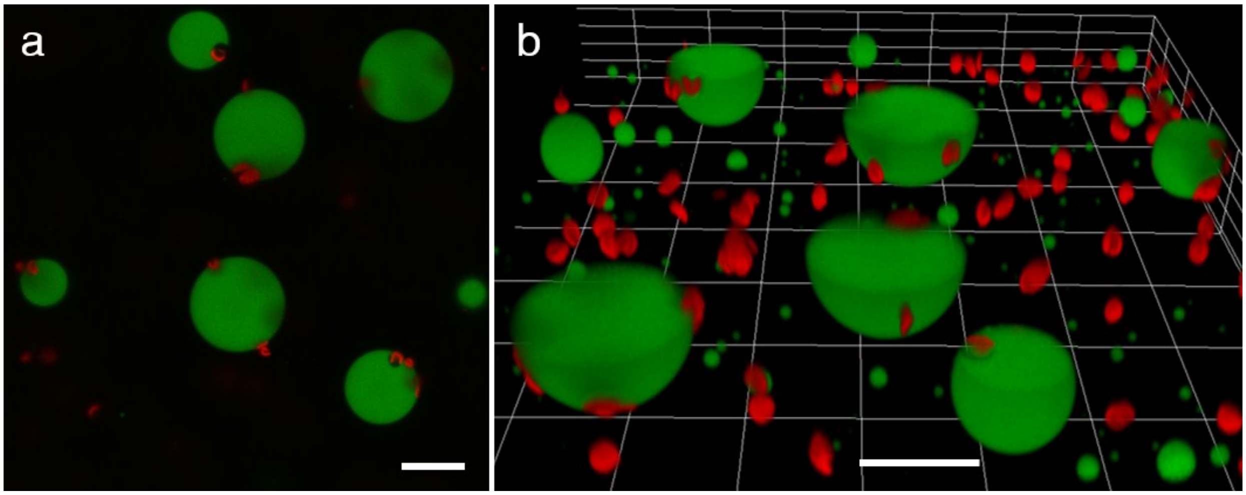

Figure S7. Confocal fluorescence microscopy images (a,b) showing capture of chloroplasts at the surface of PDDA/CMDX (5 % FITC-CMDX) coacervate micro-droplets. Samples were prepared by allowing the micro-droplets to settle onto a PEGylated glass coverslip of a custom-built sample holder followed by addition of a chloroplast dispersion to the coverslip. Scale bars, 20 µm.

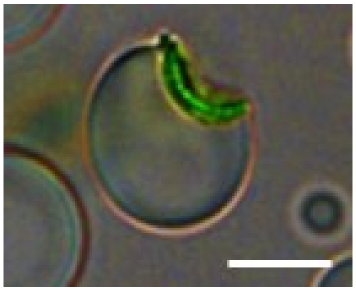

Figure S8. Optical microscopy image showing a positively-charged PDDA/CMDX coacervate micro-droplet and surface-attached negatively charged chloroplast. The surface of the coacervate droplet is distorted due to conformal wetting associated with strong electrostatic interactions between the colloidal objects. Scale bar, 5 µm.

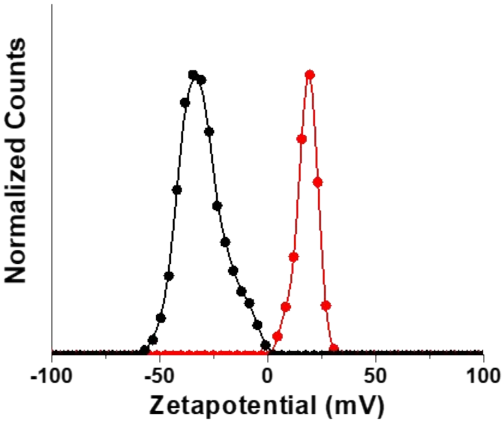

Figure S9. Zeta potential measurements of PDDA/CMDX coacervate dispersions prepared at an initial monomer mole ratio of 1 : 20 (black) or 0.3 : 1 (red) showing values of *ca.* -30 mV and + 20 mV respectively.

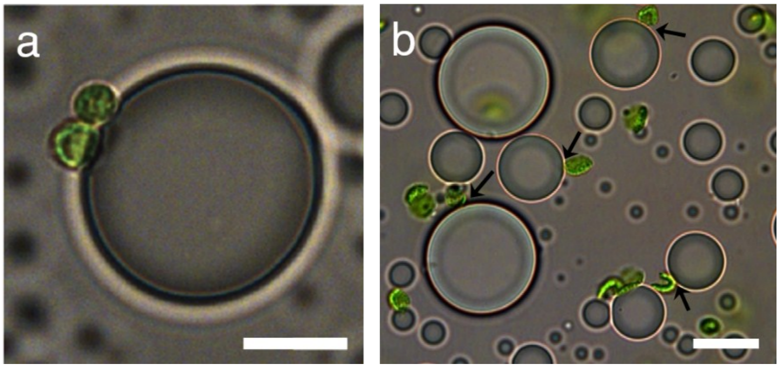

Figure S10. Bright field microscopy images (a,b) showing minimal interactions between negatively charged PDDA/CMDX coacervate micro-droplets (PDDA/CMDX molar ratio of 1 : 20) and negatively charged chloroplasts. Wetting was not observed even in contact areas between the chloroplasts and coacervate micro-droplets (arrows in b). All scale bars, 10 µm.

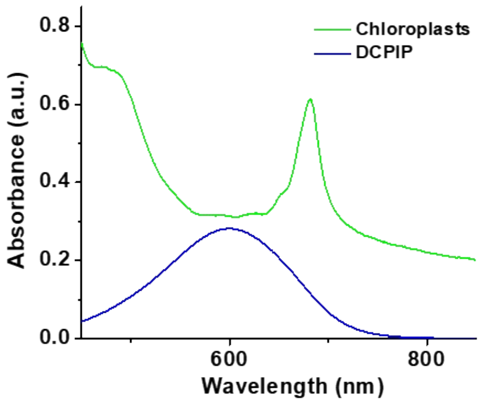

Figure S11. Absorption spectra of aqueous $DPIP_{ox}$ (blue) and an aqueous buffered suspension of chloroplasts (green) showing mutually exclusive absorption peaks. Decreases in the intensity of the $DPIP_{ox}$ peak at around 600 nm was used to monitor the formation of $DPIP_{red}$ during photosynthetic activity of the chloroplasts.

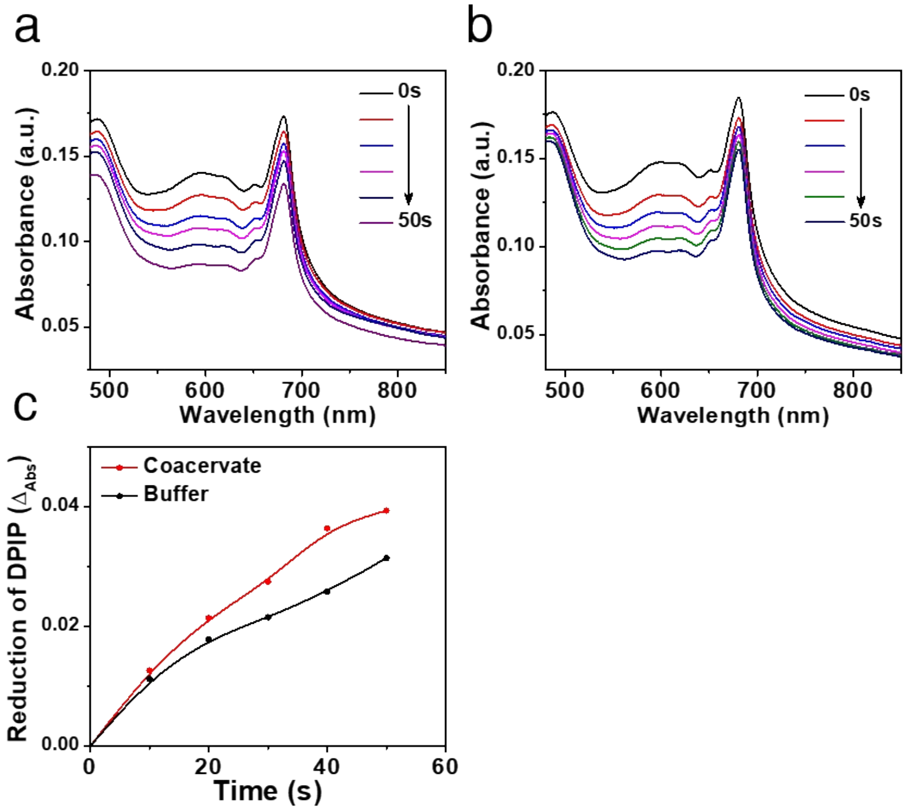

Figure S12. Time-dependent sequence of $DPIP_{ox}$ absorption spectra recorded on chloroplast-containing PDDA/CMDX coacervate micro-droplets **(a),** and in buffer in the absence of coacervates **(b)** under intermittent light exposure**.** Aliquots were analysed by UV-spectroscopy after brief exposures (10 s) of the dispersions to 40 kilolux of light. Dispersions were stored in the dark on an ice bath between light exposures. **(c)** Plot showing reduction of DPIP with time due to photosynthetic activity of chloroplasts sequestered into PDDA/CMDX coacervate droplets (red) or as free chloroplasts dispersed in buffer (black).

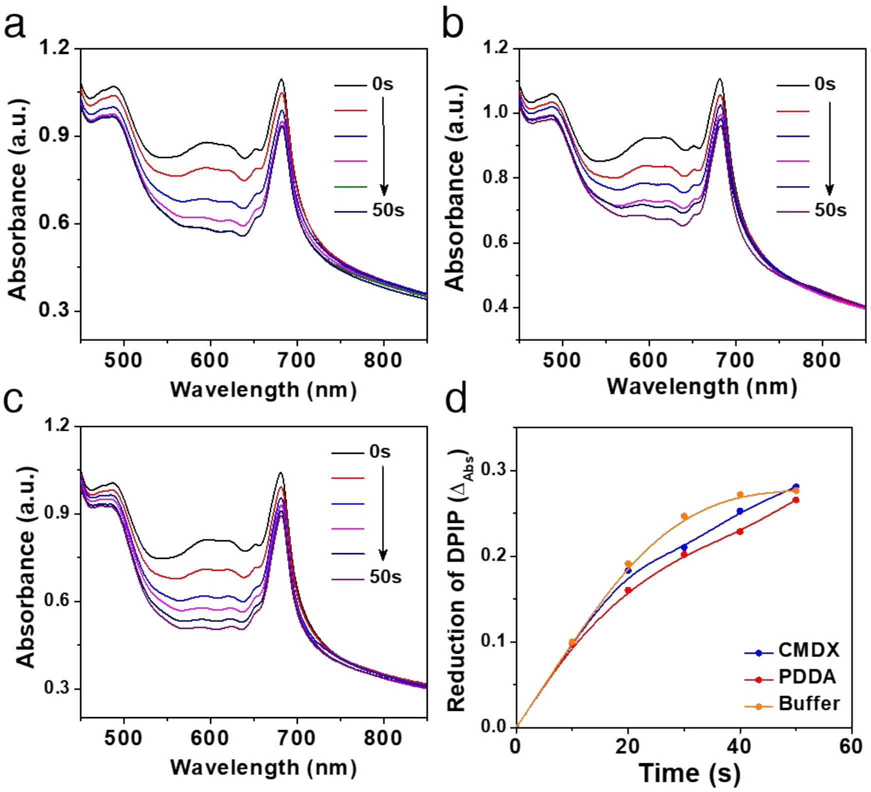

Figure S13. Time-dependent sequence of DPIP$_{ox}$ absorption spectra recorded on free chloroplasts in buffer **(a)**, in the presence PDDA in buffer **(b),** and in the presence of CMDX in buffer **(c)** under intermittent light exposure. **(d)** **(c)** Plot showing reduction of DPIP with time due to photosynthetic activity of free chloroplasts dispersed in buffer (orange), buffered PDDA (red) or buffered CMDX (blue). In each case the photosynthetic activity is essentially the same.

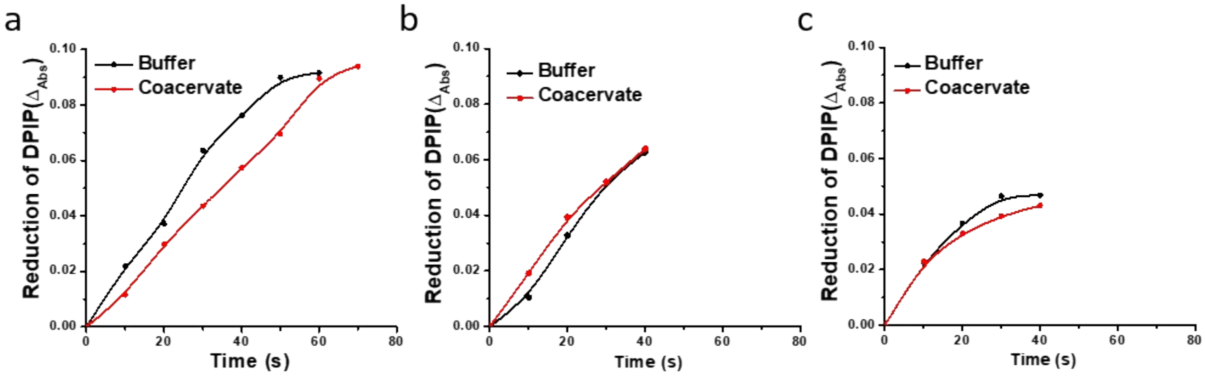

Figure S14. Plots showing reduction of DPIP with time associated with the photosynthetic activity of chloroplasts (22.4 µg/mL) internalized into PDDA/CMDX coacervate micro-droplets and dispersed in buffer in the absence of coacervate using a total concentration of (a) 60 µM, (b) 45 µM, or (c) 30 µM of DPIP in the assay.

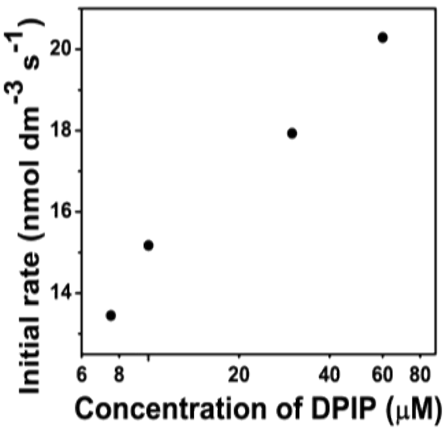

Figure S15. Plot of initial rate of the Hill reaction with respect to the concentration of $DPIP_{ox}$ showing a weak dependence on the concentration of $DPIP_{ox}$.

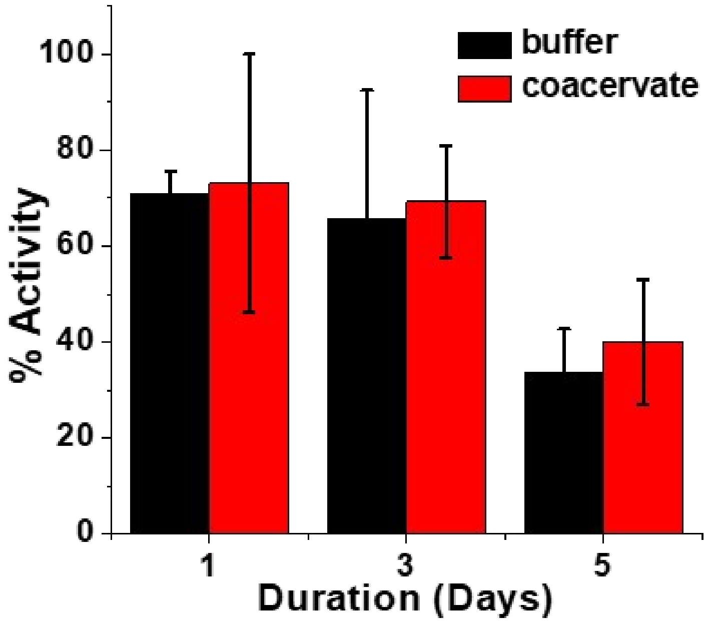

Figure S16. Degradation of photosynthetic activity of chloroplasts internalized into coacervate micro-droplets or suspended in buffer over a period of five days showing similar decay profiles with a half-life period of around 3 days.

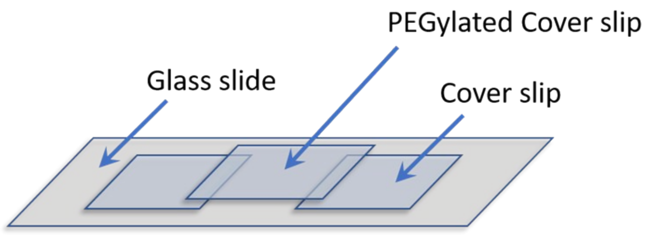

Figure S17. Sample holder used for optical and fluorescence microscopy imaging. Two cover slips were adhered to one side of a microscope slide with a channel or space between them, which was then enclosed by fixing a PEGylated cover slip on top using a UV-responsive glue. The coacervate micro-droplet dispersions were introduced into the channel from one of the open sides, and the sample holder was inverted to allow the coacervates to settle onto the PEGylated coverslip. The chloroplast dispersion was then introduced from the other side of the chamber and the interaction between the two dispersions observed using optical and fluorescence microscopy.

References:

1. R. Höinghaus and J. Feierabend, *Protoplasma*, 1983, **118**, 114-120.
2. D. I. Arnon, *Plant Physiol.*, 1949, **24**, 1-15.
